# Exendin-4 Protects MIN6 Cells from t-BHP-Induced Apoptosis via IRE1-JNK-Caspase-3 Signaling

**DOI:** 10.1155/2012/549081

**Published:** 2012-03-18

**Authors:** Wen-Jia Chen, Lin-Xi Wang, Yan-Ping Wang, Zhou Chen, Xiao-Ying Liu, Xiao-Hong Liu, Li-Bin Liu

**Affiliations:** Department of Endocrinology, Fujian Institute of Endocrinology, Union Hospital of Fujian Medical University, Fuzhou, Fujian 350001, China

## Abstract

*Objectives.* This study aimed to explore the effect of exendin-4 on t-BHP-induced apoptosis in pancreatic **β** cells and the mechanism of action. *Methods.* Murine MIN6 pancreatic **β** cells were treated with exendin-4 in the presence or absence of tert-butyl hydroperoxide (t-BHP). Cell survival was assessed by MTT staining. The percentage of apoptotic cells was determined by fluorescence microscopy analysis after Hoechst/PI staining and flow cytometric assay after Annexin V-FITC/PI staining. The activity of caspase-3 was determined using a caspase-3 activity kit. Expression of P-IRE1**α**, IRE1**α**, C-Jun N-terminal kinase (JNK), P-JNK, C-JUN, and P-C-JUN was detected by western blotting. *Results.* Exendin-4 was found to inhibit t-BHP-induced apoptosis in pancreatic **β**-cells by downregulating caspase-3 activity. Exendin-4 also inhibited the endoplasmic reticulum transmembrane protein IRE1, the apoptosis-related signaling molecule JNK, and c-Jun activation. *Conclusions.* Our findings suggest that exendin-4 ultimately reduces t-BHP-induced **β**-cell apoptosis. IRE1-JNK-c-Jun signaling is involved in the exendin-4-mediated modulation of **β**-cell apoptosis.

## 1. Introduction

Type 2 diabetes is caused by complex interactions between insulin resistance in the peripheral tissues and impaired insulin secretion by pancreatic *β*-cells. There is a general consensus that the latter results from both impaired *β*-cell function and decreased *β*-cell mass. The high activity of molecules, such as reactive oxygen species (ROS) and clusters of reactive nitrogen species (RNS), could cause oxidative damage, leading to tissue injury. The classical pathway of apoptosis includes the cell death receptor pathway (extrinsic pathway) and the mitochondrial death pathway (intrinsic pathway). Recent studies have revealed that the endoplasmic reticulum (ER) is an organelle that can sense various stresses and transmit apoptotic signals [1, 2]. One characteristic feature of *β*-cells is a highly developed ER, which arises from the large amounts of insulin secretion [3]. Abnormal oxidation and impaired protein folding can lead to endoplasmic reticulum stress (ERS).

Glucagon-like peptide 1 (GLP-1), which is secreted in a glucose-dependent manner, is involved in glucose-stimulated insulin secretion, insulin biosynthesis, inhibition of glucagon secretion and gastric emptying, and the inhibition of food intake. GLP-1 also inhibits *β*-cell apoptosis and promotes *β*-cell proliferation in animals and cultured cells in vitro. The chronic administration of GLP-1 also promotes insulin synthesis, *β*-cell proliferation, and *β*-cell neogenesis [4–6]. An important locus for the regulation of GLP-1 biological activity is the N-terminal of the peptide via dipeptidyl peptidase IV (DPP-4)-mediated cleavage at the position 2 alanine. The half-life of active GLP-1 in the circulation is only approximately 2 min, which limits its clinical value. Exendin-4 is a GLP-1 receptor agonist that is not cleaved by DPP-4. Therefore, it has a longer half-life than GLP-1 and would be more suitable as a therapeutic agent [7]. 

 At present, the action of GLP-1 on the ERS signaling pathway in pancreatic *β* cells has not been fully explained. Yusta et al. [8] demonstrated that GLP-1 receptor signaling directly modulates the ER stress response, leading to the promotion of *β*-cell adaptation and survival. Ferdaoussi et al. [9] found that exendin-4 inhibits apoptosis elicited by IL-1, which highlights the importance of GLP-1 mimetics as new potent inhibitors of cytokine-induced JNK signaling.

Tert-butyl hydroperoxide (t-BHP) is an organic lipid hydroperoxide analog, which is commonly used as a pro-oxidant to evaluate mechanisms involving oxidative stress in cells and tissues [10]. In this study, we investigated whether t-BHP can lead to ERS. Furthermore, we investigated whether exendin-4 could protect *β* cells from t-BHP-induced apoptosis. Moreover, we explored the antiapoptotic molecular mechanisms of exendin-4, including an assessment of the ERS and JNK signaling pathways, in t-BHP-treated *β* cells.

## 2. Materials and Methods

### 2.1. Reagents

Exendin-4, t-BHP, Dulbecco's modified Eagle's medium (DMEM), Hanks' balanced salt solution (HBSS), and fetal bovine serum (FBS) were obtained from Gibco (Grand Island, NY, USA). Primary antibodies, including rabbit polyclonal antibodies to sheep P-IRE1*α* and IRE-1*α*, were purchased from Santa Cruz Biotechnology (Santa Cruz, CA, USA). Rabbit polyclonal antibodies to sheep NH2-terminal kinase (JNK), p-JNK, c-Jun, p-c-Jun, caspase-3 were purchased from Cell Signaling (Beverly, MA, USA). The JNK inhibitor, SP600125, was purchased from Invitrogen (Invitrogen, Carlsbad, CA). Hoechst 33342/PI, caspase-3 activity assay kits, and the Annexin V-FITC apoptosis kit were purchased from Sigma-Aldrich (St. Louis, MO, USA). The western blot chemiluminescent detection system (LumiGLO system) was purchased from KPL (Gaithersburg, MD, USA). All reagents were of analytical or cell culture grade purity.

### 2.2. Cell Culture

The pancreatic MIN6 *β*-cell line was a gift from the Institute of Endocrinology of Ruijin Hospital, which is affiliated with Shanghai 2nd Medical University (Shanghai, China). MIN6 cells were maintained in DMEM supplemented with 15% FBS, 100 units/mL penicillin, and 100 *μ*g/mL streptomycin and were kept at 37°C in humidified air with 5% CO_2_. The cells were grown up to 75% confluence and passaged every 3 days.

### 2.3. Hoechst 33342/PI Staining

Cells were double-stained with Hoechst 33342 and propidium iodide (PI) to distinguish apoptotic cells from necrotic cells. Cells were treated with t-BHP (25 *μ*M) with or without exendin-4 (100 nM) for the indicated time, washed with PBS (pH 7.4), and then stained with Hoechst 33342 (10 *μ*g/mL) and PI (10 *μ*g/mL) for 5 min at room temperature. One hundred cells were selected at three independent times and counted under a fluorescence microscope, and the rate of apoptosis was then calculated.

### 2.4. Annexin V/PI Assay

Annexin V-FITC binding and PI staining were performed according to the manufacturer's protocol and then analyzed by flow cytometry (FACScan, Becton Dickinson). Apoptotic cells were defined as the population that were PI negative (indicating an intact plasma membrane) and Annexin V-FITC positive.

### 2.5. Caspase-3 Activity Assay

The caspase-3 assay was performed according to the manufacturer's protocol. Briefly treated cells were washed once with ice-cold PBS and assayed for caspase-3 activity using a colorimetric assay. Cleavage of Ac-DEVD-pNA substrate by caspase-3 releases pNA, which was quantified spectrophotometrically at 405 nm using an ELISA reader. The change in optical density is directly proportional to caspase-3 activity.

### 2.6. Western Blot Analysis

The treated cells were rinsed with ice-cold PBS and then incubated with RIPA lysis buffer containing 50 mM Tris-HCl (pH 7.4), 150 mM NaCl, 1% Triton-X 100, 1 mM EDTA, 1 mM NaF, 1 mM Na_3_VO_4_, 0.1% SDS, 0.5% (w/v) sodium deoxycholate, 1 mM phenylmethanesulfonylfluoride (PMSF), 10 *μ*g/mL aprotinin, 1 *μ*g/mL leupeptin, and 1 *μ*g/mL pepstatin for 20 min. The cell lysates were then centrifuged at 12,000× g for 10 min, and the protein concentrations were determined using the Bradford method. Total cell protein (50 *μ*g) was separated by 8% or 12% sodium dodecyl sulfate-polyacrylamide gel electrophoresis (SDS-PAGE) and transferred to PVDF membranes. The membranes were incubated with the following appropriate primary antibodies: P-IRE1*α* (1 : 1,000), IRE-1*α* (1 : 1,000), JNK (1 : 1,000), p-JNK (1 : 1000), c-Jun (1 : 1,000), p-c-Jun (1 : 1,000), caspase-3 (1 : 1,000). Secondary horseradish peroxidase-conjugated antibody detection was performed with enhanced chemiluminescence reagents. Quantification of the band density was performed by densitometric analysis.

### 2.7. Statistical Analysis

Data were analyzed by SigmaStat 3.5 software and shown by the mean ± standard deviation (SD) of at least three independent experiments. Statistical differences between values were determined by Student's *t*-test or ANOVA followed by Tukey's post hoc test. The significance level was set at *P* < 0.05.

## 3. Results

### 3.1. Exendin-4 Inhibits t-BHP-Induced *β*-Cell Apoptosis

**Figure 1:**
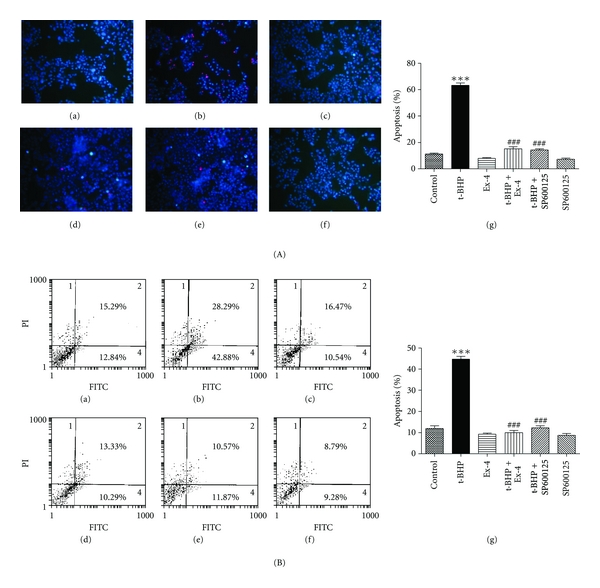
Exendin-4 inhibits t-BHP-induced *β*-cell apoptosis. MIN6 cells were preincubated with exendin-4 (100 nM) or with SP600125 (10 nM) for 18 h and then exposed to t-BHP (25 *μ*M) for 1 h. The rate of apoptosis was determined by Hoechst/PI staining (A) and flow cytometry (B). The cells containing bright blue particles (apoptosis-positive cells) were visualized under a light microscope (A). (a) Control; (b) t-BHP alone; (c) exendin-4 (Ex-4) alone; (d) Ex-4 + t-BHP; (e) SP600125 + t-BHP; (f) SP600125; (g) quantification of apoptotic cells. Values are expressed as the mean ± SD (*n* = 3). ***P < 0.001  compared with the control group; ^###^ *P* < 0.001  versus t-BHP alone.

The treatment of *β*-cells with 25 *μ*mol/L t-BHP produced the maximal apoptotic response after 1 h as evidenced by results of the Hoechst/PI and Annexin V-FITC/PI assays (data not shown). *β* cells treated with 25 *μ*mol/L t-BHP for 1 h clearly exhibited staining that was indicative of apoptosis (bright blue particles) (Figure 1(A)(b)). Interestingly, exendin-4 treatment markedly inhibited the apoptotic bright blue particle formation in MIN6 cells (Figure 1(A)(d)). An Annexin V-FITC/PI quantification assay demonstrated that t-BHP-induced MIN6 cell death was mediated by apoptosis (Figure 1(B)(b)) and that exendin-4 protected MIN6 cells from t-BHP-induced apoptosis (Figure 1(B)(d)). The inhibitory effect of exendin-4 was 77.6% (*P* < 0.001), whereas JNK inhibitor produced a 72.5% (*P* < 0.001) reduction in the level of apoptosis induced by t-BHP (Figure 1(B)(e)), which suggested that JNK signaling is involved in this process.

### 3.2. Exendin-4 Inhibits t-BHP-Induced Caspase-3 Activity

**Figure 2:**
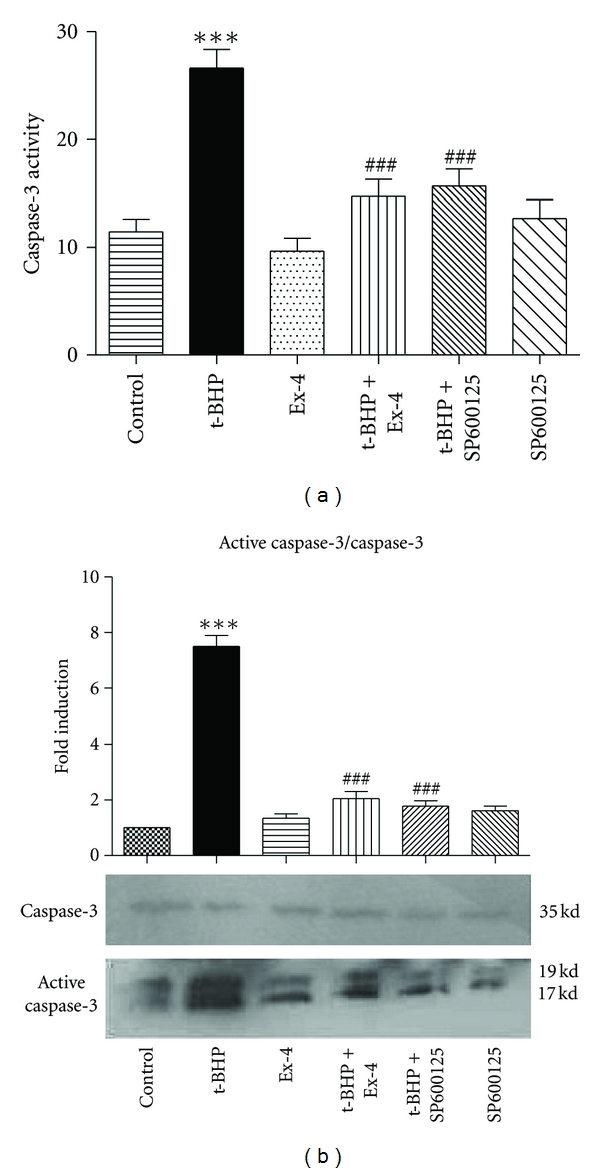
Exendin-4 inhibits caspase-3 activity in t-BHP-treated MIN6 cells. MIN6 cells were preincubated with exendin-4 (100 nM) or with SP600125 (10 nM) for 18 h and then exposed to t-BHP (25 *μ*M) for 1 h. Caspase-3 activity was determined using a caspase-3 activity assay (a) and western blotting (b), as described in Section 2. The histogram shows the quantification of the protein data. Levels of active caspase-3 protein were normalized to caspase-3 levels and expressed as the relative fold change compared to the control samples. Values are expressed as the mean ± SD (*n* = 3). ***P < 0.001 versus control group; ^###^ *P* < 0.001 versus the t-BHP group.

As shown in Figures 2(a) and 2(b), exposure of MIN6 cells to 25 *μ*mol/L t-BHP for 1 h resulted in approximate 2.3-fold Figure 2(a) and 7.5-fold Figure 2(b) (*P* < 0.001) increases in activity of the prototypic apoptotic marker caspase-3. Pretreatment of cells with exendin-4 reduced caspase-3 activity levels to 44.7% Figure 2(a) and 72.8% Figure 2(b) lower than that observed in the group treated with t-BHP alone (*P* < 0.001). This was similar to the protective effect of the JNK inhibitor, SP600125. These results suggest that exendin-4 can attenuate t-BHP-induced apoptotic death by inhibiting the activation of caspase-3 in *β* cells and that JNK signaling is involved. 

### 3.3. Exendin-4 Inhibits t-BHP-Induced Increase in IRE

**Figure 3:**
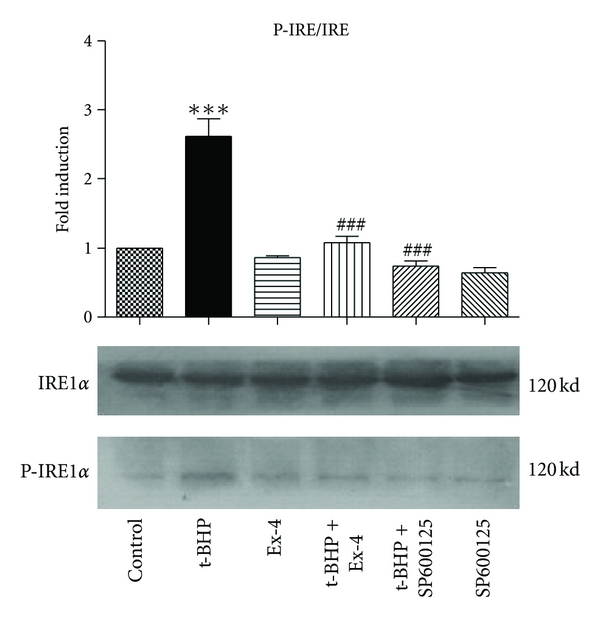
Exendin-4 inhibits t-BHP-induced increase in IRE1. MIN6 cells were preincubated with exendin-4 (100 nM) or with SP600125 (10 nM) for 18 h and then exposed to t-BHP (25 *μ*M) for 1 h. Representative western blot images revealed the expression levels of phospho-IRE and total IRE. The histogram shows the quantification of the protein data. Levels of phosphorylated protein were normalized to the levels of total protein and expressed as the relative fold change compared to the control samples. Values correspond to the mean ± SD (*n* = 3). ***P < 0.001  compared with the control group; ^###^ *P* < 0.001  versus t-BHP alone.

IRE1 is one of the three ER transmembrane proteins. Western blot analysis showed that t-BHP increases IRE1 phosphorylation by 2.6-fold (*P* < 0.001) relative to the control group (Figure 3). Pretreatment of cells with exendin-4 reduced the t-BHP-induced increase in IRE phosphorylation by 58.7% (*P* < 0.001) compared to the t-BHP alone group. This was similar to the protective effect of the JNK inhibitor, SP600125. These results indicated that ERS is probably required for the apoptotic events mediated by t-BHP and that JNK signaling is involved.

### 3.4. Exendin-4 Inhibits t-BHP-Induced Apoptosis via the JNK Signaling Pathway

**Figure 4:**
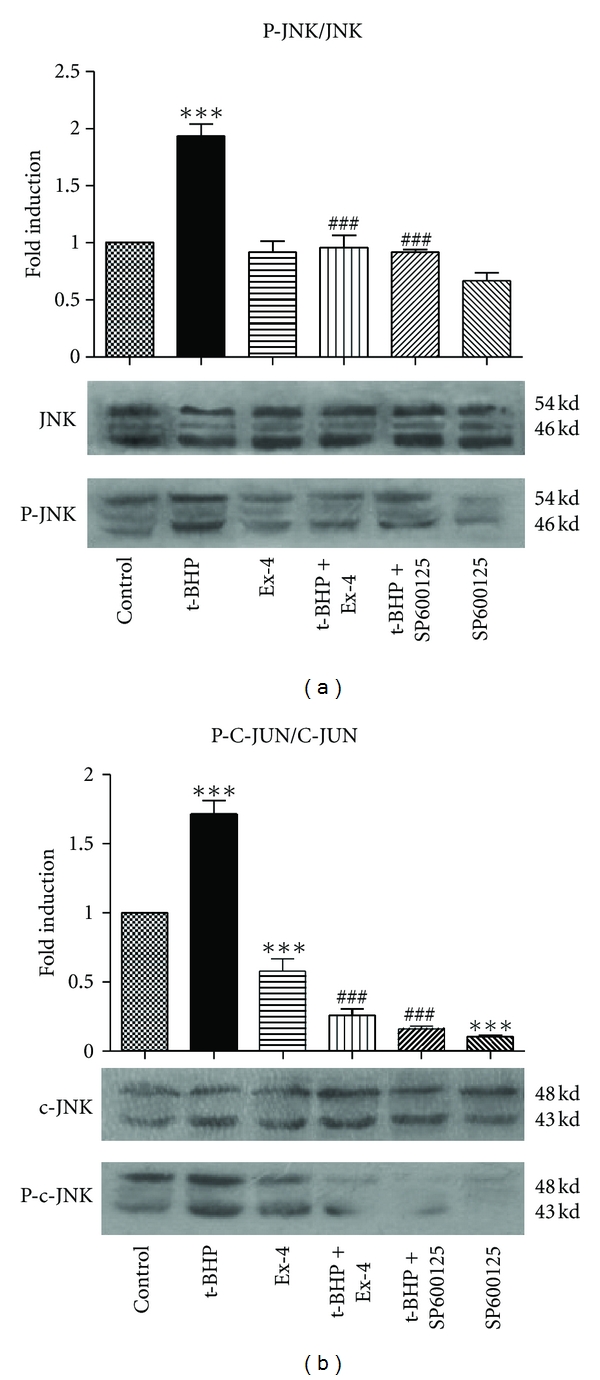
Exendin-4 inhibits t-BHP-induced aptosis via* JNK signaling pathway*. MIN6 cells were preincubated with exendin-4 (100 nM) or with SP600125 (10 nM) for 18 h and then exposed to t-BHP (25 *μ*M) for 1 h. Representative western blot images revealed the expression levels of phospho-JNK and total JNK protein (a); phospho-c-JUN and total c-JUN (b). The histogram shows the quantification of the protein data. Levels of phosphorylated protein were normalized to the levels of total protein and expressed as the relative fold change compared to the control samples. Values correspond to the mean ± SD (*n* = 3). ***P < 0.001 compared with the control group; ^###^ *P* < 0.001 versus t-BHP alone.

It is well known that the accumulation of proteins in the lumen of the ER initiates a stress response known as the unfolded protein response (UPR)/endoplasmic reticulum overload response (EOR). One of the pathways activated after ERS is the SAPK/JNK pathway. Further experiments showed that t-BHP increases JNK phosphorylation by 1.9-fold (*P* < 0.001) (Figure 4(a)) and c-Jun phosphorylation by 1.7-fold (*P* < 0.001) (Figure 4(b)). Pretreatment of cells with exendin-4 reduced the t-BHP-induced increase in JNK phosphorylation by 50.4% (*P* < 0.001) (Figure 4(a)) and reduced the t-BHP-induced increase in c-Jun by 84.9% (*P* < 0.001) (Figure 4(b)). These results suggest that exendin-4 attenuates t-BHP-induced apoptotic death by modulating JNK-c-JUN signaling in *β* cells.

## 4. Discussion

In the present study, we investigated the effects of exendin-4 on t-BHP-induced apoptosis. We demonstrated that exendin-4 protects pancreatic *β* cells from t-BHP-induced apoptotic death via IRE1-JNK-caspase-3 signaling, which suggests the probable involvement of ER stress in apoptosis.

Type 2 diabetes is associated with a gradual loss of insulin secretion and a progressive reduction in *β*-cell mass. Insulin resistance produces a sustained increase in demand for insulin, and, over time, the *β* cells are unable to sustain the augmented levels of insulin biosynthesis and secretion. Pancreatic *β* cells are extremely sensitive to ERS. The ER has several important functions, including posttranslational modification, folding, and assembly of newly synthesized secretory proteins, and it also acts as a cellular calcium store. ERS is conducive to the maintenance of the normal function of cells and their survival; however, prolonged ERS can induce cell apoptosis. Therefore, *β*-cell apoptosis induced by chronic ERS is important in type 2 diabetes [11, 12]. In our previous studies, we demonstrated that MIN6 cell viability, when treated with t-BHP, was reduced in a dose-dependent manner. We also found that continuous exposure to t-BHP induced oxidative damage in MIN6 cells [13]. The present study suggests that t-BHP treatment leads to the activation of death effector caspases, such as caspase-3, resulting in nuclear fragmentation and apoptosis (Figures 1 and 2). Further, t-BHP might trigger apoptosis in *β* cells via ERS signaling pathways (Figure 3).

IRE1 is one of the three ER transmembrane proteins. A small fragment of the X-box binding protein 1 (XBP1) mRNA is spliced out by the active form of IRE1 to produce the active form of XBP1. This is supported by the observation that the stress effect caused by IRE is mediated no later than the role of PEK-related endoplasmic reticulum eukaryotic initiation factor 2*α* kinase (PERK) and activating transcription factor 6 (ATF6) [14]. We believe that IRE is the final activated molecule in the stress response. However, in response to ERS, IRE1*α* has been found to recruit the adaptor protein, TNF receptor-associated factor 2 (TRAF2), to the ER membrane. The IRE1*α*/TRAF2 complex then recruits apoptosis signal regulating kinase 1 (ASK1), causing activation of ASK1 and the downstream mitogen-activated protein kinase family (MAPKs) cascades, which leads to cell death [15].

JNK kinases (JNKs) have been extensively characterized. JNK activation occurs through phosphorylation of its amino acid residues. Once activated, JNK is translocated from the cytoplasm to the nucleus, which in turn induces phosphorylation of its target transcription factor c-Jun [16]. The ER stress-mediated apoptosis pathway finally activates the mitochondrial death pathway, leading to caspase-3 activation. Therefore, the mitochondrial death pathway plays a role in synthesis and amplification in this pathway [17]. In the present study, we observed that the JNK inhibitor, SP600125, can inhibit the activity of caspase-3 (Figure 2); t-BHP increased JNK phosphorylation by 1.9-fold (*P* < 0.001) (Figure 4(a)) and c-Jun phosphorylation by 1.7-fold (*P* < 0.001) (Figure 4(b)), suggesting that the JNK signaling pathway is involved in the oxidative damage-induced apoptosis pathway.

Exendin-4 can inhibit islet *β*-cell apoptosis induced by oxidative damage [18]. Pandey and Rizvi [10] found that when INS-1 cells were incubated with exendin-4 in the presence or absence of IL-1, GLP-1 functioned as a potential inhibitor of the JNK signaling pathway to protect cells through the activation of drug induced apoptosis. Therefore, GLP-1 receptor agonists have potentially important applications in the treatment of diabetes. In our present study, we also found that exendin-4 inhibited t-BHP-induced *β*-cell apoptosis by 77.6% (*P* < 0.001) (Figure 1(B)(d)). Pretreatment of cells with exendin-4 reduced the t-BHP-induced increase in JNK phosphorylation by 50.4% (*P* < 0.001) (Figure 4(a)) and reduced the t-BHP-induced increase in c-JUN by 84.9% (*P* < 0.001) (Figure 4(b)). These effects were similar to those observed following pretreatment with the JNK inhibitor, SP600125, suggesting that exendin-4 attenuates t-BHP-induced apoptotic death by modulating JNK-c-JUN signaling in *β* cells.

High levels of ERS lead to the apoptosis of pancreatic *β* cells [19]. The GLP-1 receptor agonist, exendin-4, protects islet *β* cells by reducing the level of ERS [20]. Exendin-4 protects *β* cells against free fatty acids via the induction of the ER chaperone BiP and the antiapoptotic protein JunB, which mediate *β*-cell survival under lipotoxic conditions [21]. We show that a certain degree of oxidative injury produces obvious ERS and that the intracytoplasmic domain of the ER transmembrane protein, IRE1*α*, undergoes self-dimerization and phosphorylation-induced activation. IRE1 activation may promote apoptosis, and exendin-4 can inhibit the activation of IRE1*α* to reduce the ERS response, thereby protecting pancreatic *β* cells.

In recent years, the protective mechanisms of GLP-1 have been elucidated. Cornu et al. [22] showed that regulation of *β*-cell numbers and functions by GLP-1 depends on the cAMP/protein kinase A-mediated induction of IGF-1R expression and the increased activity of an IGF-2/IGF-1R autocrine loop. Klinger et al. [23] demonstrated that the cAMP/protein kinase A/CREB and MAPK/ERK1/2 pathways can additively control *β*-cell proliferation, whereas Aikin et al. [24] demonstrated that PI3K/AKT suppresses the JNK pathway in islets and that this crosstalk represents an important antiapoptotic consequence of PI3K/AKT activation. Widenmaier et al. [25] found that GLP-1 suppresses p38 MAPK and JNK via Akt-mediated changes in the phosphorylation state of the apoptosis signal-regulating kinase 1 in INS-1 cells and human islets, which results in the inhibition of its activity. Thus, a variety of interactions appear to be involved in the GLP-1 protection of pancreatic *β* cells against ER stress, such as CHOP, BiP, GRP78, XBP-1, ASK1, p-elf2*α* and AP1, amongst others, which remain to be investigated.

## 5. Conclusions

The present study has demonstrated that exendin-4 has a protective effect against t-BHP-mediated *β*-cell apoptosis through the inhibition of ER stress. We have shown that IRE1-JNK-c-Jun-caspase-3 pathways are involved. However, this study has only focused on one aspect of the ER stress response. Future studies will aim to identify additional downstream events that are regulated during persistent ER stress.
